# The Risks of Being a Wallflower: Exploring Links Between Introversion, Aspects of Solitude, and Indices of Well-Being in Adolescence

**DOI:** 10.3390/bs15020108

**Published:** 2025-01-21

**Authors:** Anna Stone, Megan DeGroot, Alicia McVarnock, Tiffany Cheng, Julie C. Bowker, Robert J. Coplan

**Affiliations:** 1 Department of Psychology, Carleton University, Ottawa, ON K1S 5B6, Canada; annastone@cmail.carleton.ca (A.S.); megandegroot@cmail.carleton.ca (M.D.); aliciamcvarnock@cmail.carleton.ca (A.M.); tiffanycheng@cmail.carleton.ca (T.C.); 2 Department of Psychology, University at Buffalo, Buffalo, NY 14068, USA; jcbowker@buffalo.edu

**Keywords:** adolescence, solitude, introversion, well-being

## Abstract

The aim of the current study was to examine the unique relations between introversion and indices of well-being while accounting for aspects of solitude (i.e., time spent alone, shyness, affinity for solitude, and negative thinking while alone). Participants were *n* = 1036 adolescents (15–19 years of age, *M* = 16.19 years, *SD* = 0.58; 67% girls) who completed a series of self-report measures assessing introversion, time spent alone, negative thinking while alone, motivations for solitude (shyness, affinity for solitude), and indices of well-being (i.e., loneliness, positive/negative affect, general well-being). Overall, results from correlational analyses indicated that introversion was associated with poorer functioning across all indices of well-being. However, when controlling for aspects of solitude, results from hierarchical regression analyses indicated a complex set of associations that varied across indices of well-being. Introversion remained associated significantly and negatively with well-being and positive affect, was no longer related significantly to loneliness, and became related significantly and negatively to negative affect. Findings are discussed in terms of how personality characteristics and aspects of solitude can impact the well-being of adolescents.

## 1. Introduction

### 1.1. Adolescence

Adolescence is a transitional developmental period between late childhood and early adulthood characterized by increased time spent alone, identity development, the navigation of new intimate relationships, and the shaping of personality traits that will become more stable over the course of one’s life (Klimstra et al., 2013; Weinstein et al., 2021). As a result, personality traits are particularly important to study in adolescence (De Fruyt & Karevold, 2021), a developmental period with important implications for mental health and well-being implications (Gale et al., 2013; Roberts et al., 2006; Steinmayr et al., 2019).

The *extroversion-introversion* dimension of personality may be particularly impactful on well-being during adolescence (Steinmayr et al., 2019). Extroverted individuals are bold, outgoing, and lively compared to their introverted counterparts, who are more reflective, thoughtful, and reserved (Briggs Myers, 2022; Eysenck, 1991). Whereas extroverts tend to prefer social environments and avoid being alone, introverts are more prone to seeking solitude (Card & Skakoon-Sparling, 2023; Teppers et al., 2013). Importantly, although there are happy introverts (Hills & Argyle, 2001), there is a large and robust empirical literature indicating that, overall, introversion is negatively related to aspects of psychological well-being (Anglim et al., 2020; Buecker et al., 2020; Zelenski et al., 2021). However, introversion is also related to other characteristics and behaviors that may also impact well-being (Zelenski et al., 2021).

For example, in terms of behaviors, evidence indicates that introverts tend to spend more time alone than their more extroverted counterparts (Srivastava et al., 2008), which may reduce opportunities for social interactions that serve to boost positive mood (Sandstrom & Dunn, 2014; Sprecher et al., 2023). Introversion is also positively associated with both an affinity for solitude (i.e., the tendency to enjoy spending time alone; Borg & Willoughby, 2022) and shyness (i.e., a personality trait characterized by fear and self-consciousness in social situations; Cheek et al., 1986). Being highly motivated to seek solitude may undermine adolescents’ abilities to build meaningful bonds with others, which are conducive to promoting and of paramount importance to well-being during adolescence (Bagwell et al., 2021; Coplan et al., 2019). Notwithstanding solitude motivations, spending time alone can also promote engagement in negative patterns of thinking, such as rumination, which is negatively associated with indices of well-being (i.e., negative affect, loneliness; Hipson et al., 2021).

Although there are many theories that attempt to account for why extroverts are generally happier than introverts, additional empirical research is required to better understand the unique contributions of extroversion-introversion that account for this association (Zelenski et al., 2021). Importantly, there have only been a handful of studies that have specifically explored the correlates of introversion in adolescence (e.g., Suldo et al., 2015). Accordingly, the goal of the current study was to examine associations between introversion, aspects of solitude, and indices of well-being in the under-studied developmental period of adolescence. In particular, we sought to assess the unique associations between introversion and well-being (loneliness, positive/negative affect, general well-being) after accounting for factors related to aspects of solitude, including time spent alone, motivations for seeking solitude (e.g., shyness, affinity for solitude), and negative thinking in solitude.

### 1.2. Overview of Introversion-Extroversion

Extraversion-introversion is a core personality trait that is part of the predominant contemporary models of personality (McCrae & John, 1992; Zelenski et al., 2021). At one end of the continuum, extraversion is broadly characterized as reflecting a desire for gratification from sources outside of the self, whereas introversion denotes interest in one’s internal mental faculties (Briggs Myers, 2022; Petric, 2022). Jung (1921) first proposed that introversion-extroversion describes individual differences in energy among people, which drive behavior. He argued that introverts gain energy from *inward*-focused endeavors (e.g., reflection), whereas extroverts receive energy by engaging *outwards* with others (e.g., social encounters). Eysenck later asserted that introversion-extroversion is a singular dimension of personality and individual differences in the trait are dependent on need for cortical arousal (Eysenck, 1967). Extraverts require more arousal than introverts and, in turn, may be more likely to seek stimulation through spending time with others or engaging in exciting activities (Hills & Argyle, 2001). Introverts have a lower threshold for arousal and are more likely to engage in activities that have little stimulation, such as reading a book (Eysenck & Eysenck, 1985).

Although Jung (1921) argued that people are either dichotomously introverts or extroverts, the introversion-extroversion personality trait is a continuous dimension of personality, and people usually fall between the two extremes (Petric, 2022; Zelenski et al., 2021). The introversion-extroversion dimension of personality has been conceptualized as a *super trait*, which includes additional narrower facets that could provide a further understanding of the intricacies of the dimension (Zelenski et al., 2021). Facets of extroversion-introversion include sociability/unsociability, assertiveness/passivity, gregariousness/shyness, and risk-taking/cautiousness (Costa & McCrae, 1992; Eysenck & Eysenck, 1985; Zelenski et al., 2021).

Introversion/extroversion is a robust predictor of well-being (Busseri & Erb, 2024; Zelenski et al., 2021). For example, as compared to their more introverted counterparts, extroverts tend to experience more positive emotions, which last longer and are more intense (Hemenover, 2003; Rusting, 2001). Across studies of adults, extroversion has been found to be negatively associated with loneliness (Cacioppo et al., 2006; Vanhalst et al., 2012) and robustly positively related to happiness, resilience, subjective well-being, job satisfaction, and other indices of well-being (Anglim et al., 2020; Lun & Yeung, 2019; Soto & Tackett, 2015). Although the trait of extroversion has some overlap with the greater experience of positive emotions, there are other aspects of extroversion that are associated with happiness outside the measure of emotionality (Steel et al., 2008). Moreover, in experimental manipulations, being assigned to act in more extroverted ways (e.g., being more talkative) can make people happier compared to when they are assigned to act in introverted ways (e.g., being quiet or reserved; Margolis & Lyubomirsky, 2020). Of note, relative to knowledge about the concomitants of introversion during adulthood, considerably less is known about the implications of introversion during the adolescent developmental period.

Adolescence, typically studied as the ages of 10–18 years, is often described as a tumultuous time, characterized by a plethora of biological, cognitive, behavioral, and social developmental changes (D. Wood et al., 2018). Adolescence is a critical time for pubertal and brain development, cultivating peer relationships and avoiding peer rejection, and identity formation (Corsano et al., 2006; Griffin, 2017; Luyckx & Robitschek, 2014). It is also a time associated with developing advanced reasoning skills and agency to formulate autonomous goals and engage in task-specific endeavors, which may in turn promote positive psychological development (Napolitano et al., 2021; Wehmeyer & Shogren, 2017). Personality traits are an important contributor to shaping social, biological, and health outcomes in adolescence (Carvalho & Novo, 2014; Tackett, 2006). For example, Suldo et al. (2015) found that personality accounted for 47% of an adolescent’s total life satisfaction.

As aforementioned, little is known about the psychological concomitants of introversion during adolescence. However, consistent with findings among adults, there is some indication that introverted adolescents tend to be less happy than their more extroverted counterparts (Cheng & Furnham, 2003; Young & Bradley, 1998). Moreover, introversion during adolescence has been related negatively to general well-being, as well as self-esteem, life satisfaction, and positive affect (Butkovic et al., 2012; Ho et al., 2008; Steinmayr et al., 2019). For example, in one study of younger (*M*_age_ = 14.6 years) and older adolescents (*M*_age_ = 17.4 years), introversion was significantly and negatively related to indices of well-being (i.e., positive attitudes, self-esteem, joy in life, lack of depressed mood) and positively associated with indices of ill-being (i.e., problems/worrying, somatic complaints), even after controlling for other personality traits such as neuroticism (Lampropoulou, 2018). Introverted adolescents are also more likely to report both peer-related and parent-related loneliness (Teppers et al., 2013).

### 1.3. Introversion, Solitude, and Well-Being: Considering Conceptual Mechanisms

Several possible explanations for the negative relation between introversion and indices of well-being have been proposed. For example, it has been suggested that introverts are less likely to possess positive traits (e.g., positive emotions, energy) and skills (e.g., social competence; Argyle & Lu, 1990a, 1990b), which in turn may interfere with psychological well-being. They may also be less prone to engage in social activities, which are conducive to fostering positive relationships that promote well-being (Lampropoulou, 2018; Suldo et al., 2015; Teppers et al., 2013). However, to date, there has been limited direct empirical support for these relations (Zelenski et al., 2021), particularly in adolescence. Given the well-established linkages between introversion and solitude-seeking (Burger, 1995; Thomas et al., 2021; Thomas & Nelson, 2024; Yuan & Grühn, 2023), in the current study, we considered several plausible factors related to aspects of *solitude* that may help to account for the negative association between introversion and well-being.

### 1.4. Time Spent Alone

A relatively simple explanation for why introverts report lower well-being is that they spend too much *time alone*. Time alone can be problematic in adolescence because it limits opportunities for positive interactions with peers, which are particularly important in the face of growing social expectations and norms during this developmental period (Borg & Willoughby, 2021; Bowker et al., 2020; Coplan et al., 2015). Engaging in more social interactions also promotes the obtainment of social capital, which may open doors for future opportunities to pursue endeavors perceived as rewarding (Card & Skakoon-Sparling, 2023; Zelenski et al., 2021). Results from several studies indicate a positive association between introversion and solitude in adulthood (e.g., Burger, 1995; Thomas et al., 2021; Thomas & Nelson, 2024; Yuan & Grühn, 2023). Research indicates that introverted adolescents show less aversion to being alone (Teppers et al., 2013), but, somewhat surprisingly, our review of the literature did not uncover any studies directly examining the link between introversion and time spent alone in adolescence. Limited research has examined time spent alone in adolescence (McVarnock et al., 2023). Although solitude emerges as a context for positive development in later adolescence (R. W. Larson, 1990), generally, time spent alone appears to be associated with socio-emotional difficulties, including symptoms of anxiety and depression, low self-esteem, and lower quality friendships, during adolescence (Borg & Willoughby, 2022; Coplan et al., 2019; Hipson et al., 2021; Sette et al., 2024). However, additional research is needed to establish what role, if any, the tendency to spend time in solitude plays in the links between introversion and well-being.

### 1.5. Motivations for Seeking Solitude

Just as time alone is important, it is also important to consider introverts’ underlying motivations for seeking solitude. For example, some introverts may report lower well-being because they also tend to be *shy*. In adolescence, the personality trait of shyness is characterized by feelings of wariness and self-consciousness in social contexts, particularly in situations involving perceived social evaluation (Cheek et al., 1986). Shyness is positively associated with the personality trait of neuroticism, which is likely a result of the worrying associated with neuroticism accompanying shyness (Bratko et al., 2002). However, introversion is also strongly associated with shyness in adolescents (Kwiatkowska & Rogoza, 2019). Shyness in adolescence is associated with heightened negative affect and loneliness, lower positive affect, and a negative view of the self (Coplan et al., 2021; Zhao et al., 2018). Further, shy adolescents often have both fewer social relationships overall and fewer close relationships and are more prone to peer victimization than their more sociable counterparts (Kwiatkowska & Rogoza, 2019; Ojanen et al., 2017).

Adolescents may also seek time alone due to an affinity for solitude (Borg & Willoughby, 2022). Adolescents high in an affinity for solitude seek solitude because they enjoy and value spending time alone (Coplan et al., 2019). Results from numerous studies have indicated a positive association between introversion and affinity for solitude (and related constructs) in samples of adults (e.g., Burger, 1995; Thomas & Azmitia, 2019) and adolescents (e.g., Corsano et al., 2019; Teppers et al., 2013). Of note, affinity for solitude is typically conceptualized as a relatively ‘benign’ reason for seeking solitude in adolescence, particularly when compared to shyness (Coplan et al., 2015; Daly & Willoughby, 2020). Having an affinity for solitude can be considered adaptive in adolescence, and depending on motivations, it can be associated with psychosocial benefits (Borg & Willoughby, 2022). However, results from several studies suggest that affinity for solitude often demonstrates associations with indices of socio-emotional difficulties (e.g., symptoms of anxiety and depression, low self-esteem, emotion dysregulation, loneliness) in adolescence (Borg & Willoughby, 2022; Endo et al., 2017; Wang et al., 2013), perhaps due to cascading negative social consequences of seeking solitude, for any reason, during adolescence. Mixed findings regarding affinity for solitude emphasize the need for more research during this important developmental period. Thus, motivations for solitude, such as shyness and affinity for solitude, should be considered in conjunction with introversion due to their associations with introversion and potential differential impacts on how time alone affects adolescents.

### 1.6. Negative Thinking While Alone

Finally, we considered what introverted adolescents might do during their time alone. Adolescents engage in a variety of activities while alone, which are differentially associated with adjustment (Hipson et al., 2021). One particularly maladaptive solitary activity appears to be engaging in patterns of *negative thinking*, such as worrying (Borkovec et al., 1983), rumination (Constantin et al., 2018), and procrastination (Flett et al., 2012). Introverts are characterized as individuals who spend time engaged with their inner thoughts, which may promote introspection but also patterns of negative thinking (Thomas & Nelson, 2024). In support of this notion, introversion has been positively (and moderately) associated with rumination (Conway et al., 2000; Oral & Arslan, 2017; Thomas & Nelson, 2024). However, findings pertaining to the links between introversion and negative thinking styles have been more mixed (Hui et al., 2024; Vélez et al., 2024). Results from two recent studies found that adolescents (Hipson et al., 2021) and adults (McVarnock et al., 2023) who frequently engage in thinking (e.g., rumination, daydreaming, planning) *when alone* were more likely to experience negative affect and loneliness. There is also some preliminary evidence to indicate that negative thinking styles (i.e., brooding and reflection) mediate the association between introversion and depressive symptoms (Lyon et al., 2021).

## 2. The Current Study

The goal of this research was to examine the links between introversion, aspects of solitude, and indices of well-being in the under-studied developmental period of adolescence. Further, we sought to examine whether introversion remained negatively associated with well-being after accounting for the postulated explanatory factors related to solitude, including time spent alone, motivations for seeking solitude (i.e., shyness, affinity for solitude), and negative thoughts while alone. It was hypothesized that introversion would be negatively related to general well-being and positive affect, as well as positively related to loneliness and negative affect. In terms of aspects of solitude, time alone, shyness, and negative thinking were each expected to be negatively associated with well-being. Given the aforementioned mixed findings, affinity for solitude was tentatively expected to be non-significantly (or even positively) associated with well-being. Finally, we sought to test whether associations between introversion and indices of well-being remain significant after controlling for aspects of solitude to determine whether introversion, or its known correlates, best explains variability in well-being during adolescence.

## 3. Method

### 3.1. Participants

Participants were *n* = 1036 adolescents (*n* = 304 boys, *n* = 692 girls, *n* = 40 did not specify) ages 15–19 years (*M* = 16.19, *SD* = 0.58) attending public high schools in Eastern Ontario, Canada. Participants were students in an elective *Introduction to Psychology*, *Sociology, and Anthropology* course. The initial sample consisted of 1140 participants. Participants who did not provide written consent (*n* = 29) or did not indicate their age (*n* = 75) were removed.

Although school boards did not permit the collection of individual data on ethnicity or socioeconomic status, the participating schools were from a mix of urban, suburban, and rural neighborhoods. In addition, the most recent publicly available data for the 2019–2020 academic year indicated that: (1) 73% of secondary school students lived in homes where English was the primary language; (2) 20% came from a lower-income home, 14% from a middle-income home, 38% from an upper-income home, and 19% preferred not to answer; and (3) 58% identified as White, with a variety of racial/ethnic identities represented (e.g., 14% Middle Eastern, 9% Black, 8% South Asian, 11% East Asian, 4% Southeast Asian, 3% Latino/Latina/Latinx, 2% Indigenous, 1% Other; Ottawa-Carleton District School Board OCDSB, 2020).

### 3.2. Procedure

Following approval from the university ethics board and public School boards, written parental consent and online adolescent participant consent were obtained. Participants completed the online survey during class time on a laptop or smartphone. Surveys were completed independently, but graduate students and trained research assistants were available for questions and clarification of survey items. Survey responses were anonymous. Data were collected in two cohorts: Cohort 1 (October 2022–April 2023) and Cohort 2 (November 2023–May 2024).

### 3.3. Measures

#### 3.3.1. Introversion

Introversion was assessed using the extraversion/introversion subscale of the *Big Five Personality Inventory* (John & Srivastava, 1999). The subscale contains 8 items that ask participants to rate how much each statement reflects them as a person (e.g., “generates a lot of enthusiasm”, “tends to be quiet”). Items are rated on a 5-point scale (from 1 = “disagree strongly” to 5 = “agree strongly”). Of note, to ease presentation and interpretation, extraversion scores were reverse-coded such that higher scores reflect *higher* introversion. The extraversion/introversion subscale was found to have good internal reliability in the current sample (α = 0.84).

#### 3.3.2. Time Alone

Time alone was measured by aggregating two items assessing the average number of times during the last 7 days they spent at least 15 min alone (1 = “not at all”, 6 = “more than 3 times a day”) and hours they spent alone in the last seven days (1 = “<1 h [<15 min per day]”, 6 = “more than 15 h [more than 2 h per day]” (Coplan et al., 2019, 2021). Time spent alone was defined for participants as being alone or doing something alone, not including sleeping. The items of time alone were highly correlated with each other (*r* = 0.61, *p* < 0.001).

#### 3.3.3. Affinity for Solitude

Affinity for solitude was examined using the *Affinity for Solitude Scale* (AFS; Coplan et al., 2019), which was adapted from the original *Preference for Solitude Scale* (PSS; Burger, 1995). The AFS contains 9 items (e.g., “I try to structure my day so that I always have some time to myself”; I often have a strong desire to get away by myself”) rated on a 7-point scale (from 1 = “strongly disagree” to 7 = “strongly agree”). AFS is calculated by using a composite score, where higher scores indicate greater affinity for solitude, and it was found to have had good internal reliability (α = 0.86) in the current sample.

#### 3.3.4. Shyness

Shyness was assessed using the *Cheek and Buss Shyness Scale* (Cheek & Buss, 1981). The scale contains 12 items (e.g., “I feel tense when I’m with people I don’t know well”; “It is hard for me to act natural when I am meeting new people”) rated on a 5-point scale (from 1 = “very uncharacteristic or untrue” to 5 = “very characteristic or true”). Items were averaged to create a composite score, and higher scores indicated greater shyness. The shyness scale was found to have good internal reliability in the current sample (α = 0.88).

#### 3.3.5. Negative Thinking While Alone

Finally, participants’ negative thoughts while alone were measured using a single item created for the current study. Participants were asked to indicate how often they engaged in “negative thoughts (e.g., worrying, rumination, bored, procrastinating)” while they were alone (defined as “by yourself or doing something by yourself—not including sleeping”) on a 5-point scale (from 1 = “never” to 5 = “most of the time”).

#### 3.3.6. Positive and Negative Affect

Affect was measured using the *positive and negative affect schedule* (PANAS; Watson et al., 1988). This measure includes a 10-item subscale for both positive affect (e.g., “interested”, “enthusiastic”) and negative affect (e.g., “distressed”, “guilty”). Participants could indicate their agreement using a 5-point scale (from 1 = “not at all” to 5 = “extremely”). The scales are calculated using a sum, where higher scores indicate greater positive or negative affect. Both subscales were found to have good internal reliability in the current sample (α = 0.80 for positive affect; α = 0.86 for negative affect).

#### 3.3.7. Loneliness

Loneliness was assessed using the *UCLA Loneliness Scale Short Form* (Russell, 1996). The scale includes 8 items (e.g., “I lack companionship”, “I am unhappy being so withdrawn”) that tap into how often participants experience feelings of loneliness (from 1 = “never” to 4 = “often”). The scale was found to have good internal reliability in the current sample (α = 0.83).

#### 3.3.8. Well-Being

Well-being was measured using the *EPOCH Measure of Adolescent Well-Being* (Kern et al., 2016). This measure taps into five aspects of well-being (i.e., engagement, perseverance, optimism, connectedness, and happiness). The scale contains 20 items (e.g., “I am optimistic about my future”, “there are people in my life who really care about me”) and asks participants to rate how much they believe each statement describes them (from 1 = “almost never” to 5 = “almost always”). Items were aggregated to create a general index of well-being. The scale was found to have excellent internal reliability in the current sample (α = 0.90).

## 4. Results

### 4.1. Preliminary Analyses

Descriptive statistics and inter-correlations among the main study variables are presented in Table 1. Of note, introversion was significantly related to all aspects of solitude and indices of well-being in expected directions. Specifically, introversion was positively associated with loneliness, negative affect, time alone, shyness, affinity for solitude, and negative thinking, as well as negatively associated with general well-being and positive affect.

Additionally, aspects of solitude were also generally associated with indices of well-being in the expected directions. Notably (and supporting its validity), the single-item assessment of negative thinking was positively associated with loneliness and negative affect, as well as negatively associated with well-being and positive affect. Finally, introversion was not significantly related to age, and there was no significant gender difference in introversion (*t* = −0.682, *p* = 0.496).

### 4.2. Hierarchical Regression Analyses

A series of hierarchical regressions was computed to explore the unique relations between introversion and indices of well-being (i.e., loneliness, well-being, positive affect, negative affect), while accounting for gender, time alone, motivations for seeking solitude (i.e., shyness, affinity for solitude), and negative thinking while alone. Separate equations were computed to predict each index of well-being. For each equation, gender was entered at Step 1, all aspects of solitude (shyness, affinity for solitude, time alone, negative thinking while alone) were entered at Step 2, and introversion was entered at Step 3.

Results predicting *well-being* are displayed in Table 2. At Step 2, aspects of solitude added a significant amount of explained variance in predicting well-being. Examination of individual beta values suggested that time alone, shyness, and negative thinking were negatively associated with well-being, whereas AFS was positively related to well-being. At Step 3, after accounting for these variables, introversion still displayed a significant and negative relation to general well-being.

Results predicting *positive affect* are displayed in Table 3. At Step 2, aspects of solitude added a significant amount of variance to the prediction of positive affect. Similarly to well-being, individual beta values suggested that time alone and shyness were negatively associated with positive affect, and AFS was positively related to positive affect. At Step 3, after accounting for control variables, introversion still displayed a significant and negative relation to positive affect.

Results predicting *negative affect* are displayed in Table 4. At Step 2, aspects of solitude added a significant amount of variance to the prediction of negative affect. Examination of individual beta values suggested that shyness and negative thinking most strongly predicted negative affect. However, at Step 3, after accounting for these variables, introversion demonstrated a significant *negative* association with negative affect.

Finally, results predicting *loneliness* are displayed in Table 5. At Step 2, aspects of solitude added a significant amount of variance to the prediction of loneliness. Examination of individual beta values suggested that both shyness and negative thinking most strongly predicted loneliness. However, after accounting for these variables, at Step 3, introversion was not significantly associated with loneliness.

## 5. Discussion

A large body of research has shown that introversion is negatively related to aspects of well-being across domains, time points, and cultures (Anglim et al., 2020; Gale et al., 2013; Lun & Yeung, 2019). However, only a few studies have examined the implications of introversion among samples of adolescents (e.g., Suldo et al., 2015). Moreover, it remains unclear whether introversion is associated with negative outcomes after accounting for its known correlates. Finding solace in solitude is one of the defining characteristics of introverts (Zelenski et al., 2021), but several aspects of solitude may negatively impact well-being. Accordingly, the goal of this current study was to examine the unique relation between introversion and indices of well-being in adolescence after accounting for conceptually relevant aspects of solitude (i.e., time alone, motivations for solitude, negative thinking while alone).

Overall, results from correlational analyses indicated that introversion was associated with poorer functioning across all indices of well-being. However, when controlling for aspects of solitude, results from hierarchical regression analyses indicated a complex set of associations that varied across indices of well-being. Introversion remained significantly and negatively associated with well-being and positive affect, was no longer significantly related to loneliness, and became significantly and *negatively* related to negative affect. The results from the current study speak to the complex and unique links between introversion and indices of well-being and indicate that there are other factors beyond solitary motivations, negative thinking, and time alone that account for these links in adolescence.

### 5.1. Aspects of Solitude and Well-Being in Adolescence

Although not the primary focus of the study, our results add to our understanding of how different aspects of solitude are related to well-being in adolescence. For example, shyness was positively related to loneliness and negative affect, as well as negatively related to general well-being and positive affect. These findings are consistent with previous research (Sette et al., 2024) and suggest that shyness remains a risk factor for internalizing problems in adolescence. For affinity for solitude, results supported the notion that this motivation for solitude is more benign in adolescence (Borg & Willoughby, 2021; Coplan et al., 2019). Overall, affinity for solitude was not significantly related to well-being, positive affect, or loneliness, although it did demonstrate a significant (albeit modest) positive relation with negative affect. Of note, in regressions that controlled for other aspects of solitude (i.e., shyness, time alone, negative thinking while alone), affinity for solitude demonstrated a significant and *positive* association with both general well-being and positive affect.

Both time spent alone and negative thinking while alone were positively related to loneliness and negative affect and negatively related to general well-being and positive affect. The association between time alone and indices of well-being remains relatively unexplored in adolescents, but the results are in line with previous research in adolescents (e.g., Hipson et al., 2021; R. Larson & Csikszentmihalyi, 1978), indicating that spending too much time alone can have negative implications in adolescence. Similarly, previous research has found that negative thinking in solitude can exacerbate negative emotions and lead to feelings of loneliness in adolescents (e.g., Hipson et al., 2021) and young adults (e.g., J. Lay et al., 2019; Vanhalst et al., 2012). This finding supports the notion that thinking in solitude can be an uncomfortable experience (Wilson et al., 2014).

### 5.2. Introversion, Solitude, and Well-Being

Overall, in line with predictions, results from correlational analyses indicated that introversion was significantly associated with poorer well-being indices. Specifically, introversion was negatively associated with general well-being and positive affect, as well as positively related to loneliness and negative affect. These findings are in line with results from considerable research in adults (Anglim et al., 2020; Card & Skakoon-Sparling, 2023) and add to the few studies demonstrating such effects among adolescents (Suldo et al., 2015; Teppers et al., 2013). However, when controlling aspects of solitude, results were more complex and appeared to vary across domains of well-being.

To begin, after accounting for aspects of solitude, introversion remained significantly and negatively related to adolescent general well-being and positive affect. These results suggest that aspects of solitude play a role in impacting adolescent well-being outcomes but do not account for why introverts tend to report poorer well-being. That is, our findings suggest that introverts are not less happy *because* they also tend to be more shy or spend more time alone. Rather, there seem to be aspects of the introversion-extraversion dimension of personality that explain adolescent well-being outcomes beyond aspects of solitude.

Several other potential explanations as to why introverts might experience less happiness than extroverts have been postulated (Zelenski et al., 2021). For example, extroverts may perceive ambiguous events as more positive than introverts or may have a higher set point for positive emotions (Hemenover, 2003; J. C. Lay et al., 2017). Introverts are less likely to engage in social situations and have more high-quality friendships than extroverts, which is conducive to promoting happiness (Waldinger & Schulz, 2023). Introverts may also have neurological differences in reward sensitivity, which make seeking out social situations perceived as less worthwhile (Card & Skakoon-Sparling, 2023).

Findings were more nuanced in terms of the relation between introversion and both loneliness and negative affect. After accounting for aspects of solitude, the relation between introversion and loneliness was no longer significant, and introversion evidenced a significant and *negative* association with negative affect. These findings suggest that aspects of solitude do play a potentially significant explanatory role in the links between introversion and these specific indices of adolescent ill-being.

For example, our findings indicate that the tendency to seek solitude—and spending more time alone—may help to explain why introversion displays a positive association with loneliness. Navigating new social challenges and pressures is a fundamental characteristic of adolescence; however, retreating to solitude as a respite from these stresses can lead to feelings of loneliness (Matthews et al., 2023). Our findings can be interpreted to suggest this may be particularly true for introverts (Teppers et al., 2013). Despite the belief that introverts require less social interaction than extroverts to fulfill their *need to belong* (Baumeister & Leary, 1995), emerging research indicates that social connection is equally as important for the well-being of both introverts and extroverts (Card & Skakoon-Sparling, 2023). However, this begs the question, if introverts need social connection similarly to extroverts, why are they more likely to seek solitude over spending time with others? It has been argued that introverts may *perceive* themselves to derive less benefit from spending time with others than extroverts and, in turn, find it less appealing than spending time alone (T. T. Nguyen et al., 2022; Thomas & Azmitia, 2019). However, this perception is a forecasting error, and introverts derive similar levels of happiness as extroverts from social interactions (Zelenski et al., 2013).

Forecasting errors may also be related to neurotic tendencies during solitude (i.e., shyness, negative thinking). Having a positive social mindset (i.e., motivation to engage in social interactions) is key to establishing and maintaining social relationships conducive to reducing feelings of loneliness (Turner et al., 2024). However, perceptions of having inadequate social competencies (e.g., shyness) and engaging in maladaptive coping strategies (e.g., ruminating) may negatively impact one’s mindset and, in turn, lead to feelings of loneliness (Turner et al., 2024). Importantly, the neurotic tendencies associated with aspects of solitude may have negative implications for introversions down the line. Vanhalst et al. (2012) found that at age 15, participants with the highest levels of introversion were more likely to be in a moderate decreasing trajectory of loneliness, which was associated with high levels of anxiety and social phobia at age 20. As such, it may be important to promote the use of positive solitary reappraisal strategies (e.g., highlight the benefits of solitude; Rodriguez et al., 2020; Turner et al., 2024) to buffer the negative impacts that loneliness can have on introverts.

Introversion was significantly and positively correlated with negative affect. However, after controlling for aspects of solitude in regression analyses, introversion evidenced a significant and *negative* relation with negative affect. It is possible that this result is a statistical artifact known as a suppression effect (Akinwande et al., 2015). The suppression effect is when a predictor variable increases predictive validity in other variables in a model, which impacts the ability to appropriately interpret the regression coefficients (Ludlow & Klein, 2014). As such, this finding should be interpreted with caution. Suppression effects are less replicable (Wiggins, 1973). The strong bivariate correlation between introversion and shyness suggests that the suppression effect may be present.

However, it may also be that the *neurotic* tendencies associated with aspects of solitude (i.e., shyness, negative thinking) contribute most strongly to adolescent ill-being. It is possible that when accounting for an introvert’s neurotic solitary tendencies, spending time alone reduced the negative emotions for introverts, as opposed to exacerbating them. This is in line with research that found having a capacity for solitude (i.e., feeling comfortable in solitude) is negatively related to neuroticism, suggesting that people with neurotic tendencies may experience frequent negative emotions and discomfort in solitude (Lin et al., 2020). This goes back to the notion that solitude is not inherently bad—solitude is what you make of it, which is dependent on various individualistic characteristics (Weinstein et al., 2023). Although solitude offers adolescents a place to engage in growth, self-regulation, identity development, and self-reflection (R. W. Larson, 1990; T. V. T. Nguyen et al., 2018), it may also be a place for individuals to socially withdraw and engage in rumination (Li et al., 2021).

### 5.3. Limitations and Future Directions

Although this study is relatively novel in its focus on the outcomes of introversion among adolescents, it is not without its limitations. For one, this research was cross-sectional, and thus alternative causal explanations of the relations between variables cannot be discounted—nor can the influence of other variables. For example, lonely introverts may seek out solitude because of their experiences of being ostracized (Ren et al., 2021). The cross-sectional design also prevented us from conducting formal tests of mediation, which would have allowed us to more specifically test mechanisms of influence. Understanding how aspects of solitude may impact the well-being of introverted adolescents provides initial statistical evidence of how aspects of solitude play meaningfully impact introverted adolescent’s well-being. However, future research should examine the mediating role of aspects of solitude towards further uncovering *why* introversion is associated with negative outcomes in adolescence and beyond. Also, we considered the particular developmental stage of adolescence. It is important to further examine the development and implications of introversion and aspects of solitude across the lifespan and longitudinally over time. The current study only examined individuals between mid-to-late adolescence. However, it has been argued that introversion may pose a greater risk for well-being in *early* adolescence (10–14 years), when friendships are still forming and cultivating, as opposed to late adolescence and early adulthood, when spending time alone becomes more comfortable (Vanhalst et al., 2012; Steinmayr et al., 2019) and viewed as more normative (Bowker et al., 2020; K. R. Wood et al., 2021).

From a methodological perspective, the use of a single item to assess negative thinking while alone substantially reduces the validity of this measure (Allen et al., 2022). We were also not able to consider different types of negative thinking patterns. Notwithstanding, this item did correlate in expected directions with theoretically relevant variables (e.g., shyness, negative affect), providing at least some initial support for its construct validity. However, future research will need to further explore how negative thinking impacts the relation between personality characteristics and adolescent well-being.

Finally, the current study did not include assessments of other personality traits. Previous research has suggested that the *combination* of specific traits may have heightened associations with aspects of well-being (Lynn & Steel, 2006; Steinmayr et al., 2019). For example, Young and Bradley (1998) found in a sample of adolescents that, overall, emotionally stable (i.e., less neurotic) introverts were less happy than emotionally stable extroverts. However, emotionally stable introverts were happier than emotionally unstable extroverts and introverts. As such, neurotic tendencies seem to explain some of the variance between introversion and well-being. Results from other studies also suggest that heightened neuroticism may exacerbate the negative associations between introversion and well-being (Fadda & Scalas, 2016). As such, future research will need to further explore the interactions between neuroticism and introversion (as well as other personality traits) in the prediction of well-being in adolescence.

## 6. Conclusions

A growing body of evidence has indicated the potential negative impacts that introversion can have on well-being (Butkovic et al., 2012; Ho et al., 2008; Steinmayr et al., 2019). Adolescence is a developmental time period surrounding immense transition and growth, and as such, introversion may be particularly consequential during this milestone (Corsano et al., 2006; Griffin, 2017; Luyckx & Robitschek, 2014). Yet, few studies have examined the role of introversion in adolescence. Considering that enjoyment of solitude is marked as a defining feature of introversion (Zelenski et al., 2021), the current study considered aspects of solitude as contributing factors in the relation between introversion and well-being in adolescence. Initial support was found to suggest that aspects of solitude do not play a significant role in impacting adolescent well-being or negative affect. However, the neurotic tendencies associated with aspects of solitude, such as rumination and shyness, were found to meaningfully minimize the relation between introversion and loneliness. These results highlight that while solitude may negatively impact an adolescent’s need to belong, the mechanisms that are reducing the happiness of introverts beyond aspects of solitude are still unclear. Future research will need to further explore the complex relations between personality, aspects of solitude, and well-being in adolescence to further understand under what conditions and for whom spending time alone will help versus hinder development.

## Figures and Tables

**Table 1:** Descriptive Statistics and Intercorrelations Among Study Variables.

Variables	*M*	*SD*	1	2	3	4	5	6	7	8	9	10
1. Age	16.190	0.583	-									
2. Loneliness	2.619	0.492	0.048	-								
3. Shyness	3.081	0.796	−0.025	0.299 ***	-							
4. Well-Being	3.171	0.695	0.061	−0.403 ***	−0.421 ***	-						
5. AFS	4.688	1.231	−0.029	0.013	0.268 ***	0.060	-					
6. Positive Affect	31.536	6.560	0.053	−0.198 ***	−0.396 ***	0.610 ***	0.024	-				
7. Negative Affect	27.457	8.133	−0.021	0.398 ***	0.361 ***	−0.275 ***	0.075 *	−0.019	-			
8. Time Alone	4.340	1.384	0.001	0.093 **	0.142 ***	−0.176 ***	0.195 ***	−0.164 ***	0.063 *	-		
9. Negative Thinking	3.780	1.018	−0.037	0.375 ***	0.344 ***	−0.321 ***	0.066 *	−0.205 ***	0.562 ***	0.108 ***	-	
10. Introversion	2.922	0.804	−0.010	0.165 ***	0.644 ***	−0.399 ***	0.292 ***	−0.439 ***	0.111 ***	0.172 ***	0.185 ***	-

Note: * *p* < 0.05, ** *p* < 0.01, *** *p* < 0.001. *AFS = Affinity for Solitude.*

**Table 2:** Multiple Regression Predicting Well-Being.

Variable	Model 1			Model 2			Model 3		
	*B*	*SE B*	*β*	*B*	*SE B*	*β*	*B*	*SE B*	*β*
Gender	−0.099	0.051	−0.066	0.012	0.046	0.008	−0.027	0.045	−0.018
Shyness				−0.339	0.028	−0.387 ***	−0.186	0.034	−0.212 ***
AFS				0.112	0.017	0.199 ***	0.132	0.017	0.234 ***
Time Alone				−0.069	0.015	−0.136 ***	−0.063	0.015	−0.123 ***
Negative Thinking				−0.125	0.022	−0.184 ***	−0.129	0.021	−0.190 ***
Introversion							−0.240	0.033	−0.279 ***
Adj. *R*^2^		0.003			0.247			0.290	
F for Δ*R*^2^		3.853			72.466 ***			54.114 ***	

Note: * *p* < 0.05, ** *p* < 0.01, *** *p* < 0.001. *Gender was coded Males* = 1, *Females* = 2. *AFS = Affinity for Solitude.*

**Table 3:** Multiple regression predicting positive affect.

Variable	Model 1			Model 2			Model 3		
	*B*	*SE B*	*β*	*B*	*SE B*	*β*	*B*	*SE B*	*β*
Gender	−1.683	0.465	−0.120	−1.088	0.443	−0.077 *	−1.560	0.425	−0.111 ***
Shyness				−3.247	0.271	−0.396 ***	−1.362	0.324	−0.166 ***
AFS				0.880	0.167	0.167 ***	1.111	0.161	0.211 ***
Time Alone				−0.580	0.146	−0.123 ***	−0.503	0.139	−106 ***
Negative Thinking				−0.244	0.209	−0.038	−0.294	0.199	−0.046
Introversion							−2.942	0.307	−0.365 ***
Adj. *R*^2^		0.013			0.190			0.264	
F for Δ*R*^2^		13.116 ***			50.263 ***			91.765 ***	

Note: * *p* < 0.05, ** *p* < 0.01, *** *p* < 0.001. *Gender was coded Males* = 1, *Females* = 2. *AFS = Affinity for Solitude.*

**Table 4:** Multiple regression predicting negative affect.

Variable	Model 1			Model 2			Model 3		
	*B*	*SE B*	*β*	*B*	*SE B*	*β*	*B*	*SE B*	*β*
Gender	5.068	0.562	0.287 ***	2.449	0.492	0.139 ***	2.189	0.490	0.124 ***
Shyness				1.788	0.301	0.170 ***	2.828	0.375	0.275 ***
AFS				−0.045	0.186	−0.006	0.082	0.186	0.012
Time Alone				−0.063	0.162	−0.010	−0.021	0.161	−0.003
Negative Thinking				3.769	0.232	0.479 ***	3.742	0.230	0.468 ***
Introversion							−1.623	0.355	−0.160 ***
Adj. *R*^2^		0.083			0.366			0.380	
F for Δ*R*^2^		81.319 ***			100.548 ***			20.898 ***	

Note: * *p* < 0.05, ** *p* < 0.01, *** *p* < 0.001. *Gender was coded Males* = 1, *Females* = 2. *AFS = Affinity for Solitude.*

**Table 5:** Multiple regression predicting loneliness.

Variable	Model 1			Model 2			Model 3		
	*B*	*SE B*	*β*	*B*	*SE B*	*β*	*B*	*SE B*	*β*
Gender	0.146	0.035	0.137 ***	0.045	0.034	0.042	0.042	0.034	0.040
Shyness				0.130	0.021	0.209 ***	0.139	0.026	0.223 ***
AFS				−0.024	0.013	−0.061	−0.023	0.013	−0.058
Time Alone				0.018	0.011	0.050	0.018	0.011	0.051
Negative Thinking				0.139	0.016	0.285 ***	0.138	0.016	0.285 ***
Introversion							−0.013	0.025	−0.022
Adj. *R*^2^		0.018			0.172			0.171	
F for Δ*R*^2^		17.126 ***			42.644 ***			0.295	

Note: * *p* < 0.05, ** *p* < 0.01, *** *p* < 0.001. *Gender was coded Males* = 1, *Females* = 2. *AFS = Affinity for Solitude.*

## Data Availability

Data is available upon request from the author.
